# Direct Delivery of Health Promoting β-Asp-Arg Dipeptides via Stable Co-expression of Cyanophycin and the Cyanophycinase CphE241 in Tobacco Plants

**DOI:** 10.3389/fpls.2020.00842

**Published:** 2020-06-19

**Authors:** Henrik Nausch, Mandy Dorn, Andrej Frolov, Sandra Hoedtke, Petra Wolf, Inge Broer

**Affiliations:** ^1^Department of Agrobiotechnology and Risk Assessment for Bio- und Gene Technology, Faculty of Agricultural and Environmental Sciences, University of Rostock, Rostock, Germany; ^2^Department of Bioorganic Chemistry, Leibniz Institute of Plant Biochemistry, Halle (Saale), Germany; ^3^Department of Biochemistry, Saint Petersburg State University, Saint Petersburg, Russia; ^4^Department of Nutrition Physiology and Animal Nutrition, Faculty of Agricultural and Environmental Sciences, University of Rostock, Rostock, Germany

**Keywords:** *Nicotiana tabacum*, cyanophycin, co-expression, cyanophycinase CphE, β-Asp-Arg dipeptides

## Abstract

Feed supplementation with β-arginine-aspartate dipeptides (β-Asp-Arg DP) shows growth promoting effects in feeding trials with fish and might also be beneficial for pig and poultry farming. Currently, these DPs are generated from purified cyanophycin (CGP), with the help of the CGP-degrading enzyme cyanophycinase (CGPase). As alternative to an *in vitro* production, the DPs might be directly produced in feed crops. We already demonstrated that CGP can be produced in plastids of tobacco and potato, yielding up to 9.4% of the dry weight (DW). We also transiently co-expressed CGPases in the cytosol without degrading CGP in intact cells, while degradation occurs in the homogenized plant tissue. However, transient co-expression is not feasible for field-grown CGP plants, which is necessary for bulk production. In the present study, we proved that stable co-expression of the CGPase CphE241 in CGP-producing tobacco is sufficient to degrade 2.0% CGP/DW nearly completely within 3 h after homogenization of the leaves. In intact senescing leaves, CGP is partially released to the cytosol and degraded into DPs which limits the overall accumulation of CGP but not the level of the stable DPs. Even after 48 h, 54 μmol β-Asp-Arg DP/g DW could be detected in the extract, which correspond to 1.5% DP/DW and represents 84% of the expected amount. Thus, we developed a system for the production of β-Asp-Arg DP in field-grown plants.

## Introduction

The supply of feed with sufficient amounts of (conditionally) essential amino acids (AA) such as lysine (Lys), arginine (Arg), tryptophan (Trp) or methionine (Met) is one of the major challenges in livestock production (Ufaz and Galili, 2008). Since they are underrepresented in plant-based animal diets, feed is supplemented with AA that are chemically synthesized or produced via microbial fermentation (FAO, 2004; BMBF, 2007). Beyond their nutritional value as building blocks for proteins, many AAs provide also physiological benefits, e.g. immunomodulatory effects, which promote health and wellbeing (Wu et al., 2009, 2014). In this respect, Arg proved to be unique and even though not essential in adult animals such as pigs, poultry, and fish, dietary deficiency of Arg can result in metabolic, neurological or reproductive dysfunctions (Wu, 2009; Wu et al., 2009). However, free AAs inhibit each other’s uptake, which confines their use as feed additive^1^. In contrast, di-, tri- and oligopeptides do not undergo such restrictions. In addition, dipeptides (DP) are resorbed more efficiently compared to free mono AA due to the presence of a specific di- and tripeptide specific transporters in the enterocytes of the small intestine that show a higher substrate affinity compared to transporters for free AA (Webb, 1986; Chen et al., 1999; Klang et al., 2005; Lu and Klaassen, 2006; Broer, 2008; Sallam and Steinbuchel, 2010; Santos et al., 2012; Terova et al., 2013; Hou et al., 2017). The beneficial effects of arginine-aspartate (β-Asp-Arg) and lysine-aspartate (β-Asp-Lys) DP were demonstrated in a long-term feeding trial with fish in which an increased body weight gain has been observed (Cysal, personal communication). Currently, both types of DPs are derived from the polypeptide Cyanophycin (CGP), produced in transgenic *Escherichia coli* (*E. coli*).

Cyanophycin is naturally produced in cyanobacterial and non-cyanobacterial prokaryotes as transient storage for carbon, nitrogen and energy (Frommeyer et al., 2014). It is synthesized by the CGP-synthetases (CphAs) via non-ribosomal protein biosynthesis in two steps: (1) the α-amino group of arginine is linked to the β-carboxylate group of aspartate via a β-iso-peptide bond to a β-arginine-aspartate dipeptides (β-Asp-Arg DP) with a molecular weight of 0.2 kDa, (2) the DP is subsequently connected to the nascent CGP polypeptide by an α-peptide bond. CGP aggregates to intracellular membraneless granules (Law et al., 2009). In addition to Arg, other AAs such as Lys are incorporated as well in lower amounts (Frommeyer et al., 2014). The molecular weight of the polymer is polydisperse and ranges in cyanobacteria between 25 and 100 kDa, but when expressed in *E. coli* only between 20 and 40 kDa. Due the β-peptide bond, which does not occur in proteins, CGP can only be degraded by so-called cyanophycinases (CGPase), but is resistant to common proteases (Law et al., 2009). CGPases are divided into CphB- and CphE-type enzymes, which both degrade CGP into DP monomers. While CphBs are expressed by CGP-producing prokaryotes in the cytosol and remobilize CGP in the event of starvation, CphEs are secreted by non-CGP producing prokaryotes in order to exploit CGP from other bacteria. Interestingly, CphE-expressing bacteria were found in the colon of animals as part of the natural microbiota (Sallam and Steinbuchel, 2009a). Since AA and DP uptake occurs in the small intestine (Broer, 2008; Sallam and Steinbuchel, 2009a), the produced DP cannot be absorbed by the animal.

The recombinant production of CGP has already been established in yeast, fungi or *E. coli* and cultivation conditions were optimized, increasing the accumulation level up to 40% per dry weight (DW) and promoting the incorporation of Lys (Frommeyer et al., 2014). Likewise, the CphE-type CGPase from *Pseudomonas alcaligenes* DIP-1 (CphE_al_) was expressed in *E. coli* (Sallam and Steinbuchel, 2009b; Sallam et al., 2011). This research resulted in the establishment of the Cysal GmbH (see text footnote 1). Cysal produces both CGP and the CphE-type CGPase in individual *E. coli* strains, and incubates the purified substrate with the enzyme to generate the DPs that are sold as feed additives (Sallam and Steinbuchel, 2011). However, the bioreactor-based production necessitates a complex infrastructure and is limited in terms of scalability. Hence, this system is commonly used for the synthesis of high-value, but not for cost-sensitive products such as supplements for animal diets (Merlin et al., 2014; Sabalza et al., 2014; Walwyn et al., 2015).

Alternatively, recombinant low-value products can be produced in plants in an economical manner using existing agricultural infrastructure and farming practices as modeled in case studies (Tusé et al., 2014; Tschofen et al., 2016). Studies estimated that the production costs are on average less than a tenth compared to conventional microbial and mammalian cell cultures (Broz et al., 2013; Tusé et al., 2014; Chen, 2015; Gecchele et al., 2015; Walwyn et al., 2015; Dirisala et al., 2016; Nandi et al., 2016; Buyel et al., 2017). The synthesis of CGP in plants was established via expression of the CGP-synthetase gene from *Thermosynechococcus elongatus* BP-1 (*cph*A_Te_) in plastids of stably transformed tobacco (*Nicotiana tabacum*) and potato (*Solanum tuberosum*) plants, yielding up to 9.4% leaf DW in commercial tobacco cultivars (Neumann et al., 2005; Hühns et al., 2008, 2009; Neubauer et al., 2012; Nausch et al., 2016). Plastid targeting was achieved via fusion of the coding region of *cph*A_Te_ to the transit peptide of the integral protein of photosystem II PsbY (Hühns et al., 2008). Plastidic CGP is almost exclusively composed of β-Asp-Arg DPs and has a molecular weight of 15–20 kDa. Noteworthy, the cytosolic expression yielded only low amounts of CGP and induced severe phenotypic disorders (Neumann et al., 2005).

Even though CGP-degrading prokaryotes were found in the digestive tract of animals (Sallam and Steinbuchel, 2009a), feeding of CGP-producing plants as diet might not lead to an absorption of β-Asp-Arg DPs, since the bacteria where only found in the colon, while DP uptake occurs in the small intestine (Chen et al., 1999; Klang et al., 2005; Lu and Klaassen, 2006; Broer, 2008; Terova et al., 2013). Hence, co-delivery of a CGPases would be necessary. Ideally, both substrate and degrading enzymes are produced by the same plant. To prevent premature CGP degradation and subsequent metabolizing of the resulting DPs, CGP, and CGPase need to be spatially separated. The production of both a CphB-type CGPase from *T. elongatus* BP-1 (CphB_Te_, 30.7 kDa) and a mutant of the CphE-type CGPase CphE_al_, termed as CphE241 (44.7 kDa), in the cytosol of plants was already shown via transient expression in tobacco (Ponndorf et al., 2016; Nausch and Broer, 2017). In addition, transient expression of both cytosolic enzymes in plastidic CGP-producing tobacco demonstrated that CGP was not degraded until the plant material was homogenized. Complete CGP degradation was only observed for CphE241, but not for CphB_Te_, which was assigned to a drastically higher enzyme activity of CphE241 (Ponndorf et al., 2017). In a feeding study with mice, in which purified plant-produced CGP and CphB_Te_ were added to the diet, it could be shown that the CGPase is capable to degrade CGP in the digestive tract and that the β-Asp-Arg DPs were transferred to the blood (Ponndorf et al., 2016). This should also be true for CphE_al_, since this CGPase type naturally occurs in the colon (Sallam and Steinbuchel, 2009a) and since it has been demonstrated that CphE_al_ is active in a pH range from 5 to 9 (Sallam and Steinbuchel, 2011).

Forage should be produced at large scale in the field. Hence the CGPase has to be stably expressed in the CGP-producing plants. Since yields achieved by transient expression are commonly drastically higher compared to the accumulation in stably nuclear transformed plants (Xu et al., 2012; Kopertekh and Schiemann, 2017; Schillberg et al., 2019) stable expression of CphE241 might not be sufficient to degrade CGP completely in an acceptable time frame. While expression levels up to 80% of the total soluble protein (TSP) were reported for the transient assay, corresponding to 5 mg/g fresh weight (FW), recombinant proteins do not exceed 1% of TSP in stably transformed plants, rarely exceeding 100 μg/g FW and reaching up to a maximum of 500 μg/g FW (Schillberg et al., 2019).

Here we show that the CGPase CphE241 can be stably expressed in high amounts in CGP-producing plants and that the CphE241 activity is sufficient to degrade the 2.0% CGP/DW present in the plant almost completely in 3 h. The premature release of CGP in intact older leaves into the cytosol limits the overall CGP accumulation during plant growth. However, the premature degradation does not affect the DP production, since DPs are not metabolized in plants. Conversion of 2.0% CGP/DW resulted in ∼54 μmol DP/g DW which corresponds to 14.7 mg DP/g DW or 1.5% DP/DW.

## Materials and Methods

### Construction of Plant Transformation Vector

**FIGURE 1:**
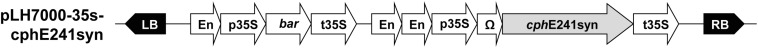
The constitutive 35S promoter is used for the expression of *cph*E241*syn* and the *bar* coding region. Schematic representation of the T-DNA region of the pLH7000-35s-cphE241syn construct used for cytosolic expression of the cyanophycinase CphE241. LB, left border; RB, right border; En, Cauliflower Mosaic Virus (CaMV) 35S enhancer; p35S, CaMV 35S core promoter; t35S, CaMV 35S terminator; Ω, Ω translational enhancer of the TMV; *bar*, coding region for the *bialaphos*-resistance protein from *Streptomyces hygroscopicus*; *cph*E241syn, codon-optimized, synthetic coding region for the CphE241 protein.

CphE241, a mutant of the CphE-type CGPase CphE_al_ (Sallam et al., 2011) encoded by *cph*E241*syn* (Nausch and Broer, 2017), was selected for stable transformation of the commercial *N. tabacum* cultivar Badischer Geudertheimer (BG) event BG 176 which produces 2–2.5% CGP/DW. *Cph*E241*syn* was transferred from the plasmid pEX-K4-CphE241syn (Nausch and Broer, 2017) into the vector pLH-IL6ER (Nausch et al., 2012). Via *Bam*HI/*Nru*I restriction/ligation, the coding region of *IL6ER* was replaced between 5′ region [consisting of the Cauliflower Mosaic Virus (CaMV) 35S promoter with double-enhancer (Odell et al., 1985), the tobacco mosaic virus (TMV) Ω translational enhancer (Gallie et al., 1987)] and the 3′ region [containing the CaMV 35S terminator (Odell et al., 1985)]. The entire gene was subsequently cloned via *Sfi*I restriction/ligation into the plant transformation vector pLH7000 [Acc. No. AY234330 (Hausmann and Toepfer, 1999)], carrying the bialaphos-resistance gene (*bar*) from *Streptomyces hygroscopicus* (Thompson et al., 1987) as selection marker, forming pLH7000-35s-cphE241syn (Figure 1) for constitutive expression. The final plasmid was verified by restriction and sequencing (Eurofins Genomics GmbH). All vectors were transferred into the *Rhizobium radiobacter* (formerly *Agrobacterium tumefaciens*) strain C58C1 for stable transformation of the *N. tabacum* event BG 176, which was already transformed with the plastid-targeted cyanophycin synthetase gene from *Thermosynecchococcus elongatus* BP-1 [*psby*-*cph*A_Te_ (Hühns et al., 2008)]. This second transformation was termed as super-transformation.

### Plant Material and Transformation

For super-transformation with the CphE241 encoding T-DNA, the T2 individual BG 176-4-3 of the one-copy event BG 176 (Nausch et al., 2016) was used. Tobacco seeds were surface sterilized in a saturated calcium hypochlorite solution, germinated on Linsmaier and Skoog (LS) medium (4.4 g/L of LS medium including vitamins (Duchefa, Haarlem, Netherlands), 30 g/L sucrose, 6.5 g/L plant agar (Duchefa), [pH 5.7]. *In vitro* grown plants were maintained at 24/22°C day/night temperature with a 16 h photoperiod. Approximately 1-month old tobacco leaves were used for *Agrobacterium*-mediated transformation as described in Wohlleben et al. (1988), using 100 μg/mL kanamycin and 20 μ/mL D/L-phosphinothricin for selection of transgenic cells. Calli/Explant and Shoot/Explant ratios were determined 6 weeks after transformation. Regenerated shoots were selected on LS medium containing 20 μg/mL D/L-phosphinothricin, 100 μg/mL kanamycin, and 500 μg/mL cefotaxim. Shoots were transferred for rooting to LS medium with herbicide and antibiotics. Total DNA was isolated from regenerated plants as described by Nausch et al. (2012) and transgene integration confirmed by PCR using CphEsyn-fw5/CphEsyn-rv1 for the coding region of *cph*E241*syn*, barPP-fw/-rv for the coding region of the selection marker, and PsbY-fw/CphA-rv1 for the *cph*A gene (Table 1).

**TABLE 1:** Primers used in this study.

**Primer**	**Sequence**	**PCR fragment size amplified by the primer pairs**	
CphEsyn-fw	5′-TGCTGCAGATGCCCAATGGATTCCC-3′	776 bp	
CphEsyn-fw5	5′-CGGTTCTTGCAGGCACCTCTGCTGG-3′		572 bp
CphEsyn-rv1	5′-AGGTTTTCTACGCCCGTTTGCGCC-3′		
PsbY-fw	5′-CCTAGCCGGAGCCGTCTTCTCTTCC-3′	585 bp	
CphA-rv1	5′-CCGGAGATCGGCGAGATCCTGATCC-3′		
barPP-fw	5′-GCACGGTCAACTTCCGTACCGAGCC-3′	461 bp	
barPP-rv	5′-ATCTCAGATCTCGGTGACGGGCAGG-3′		
Actin-fw	5′-GCAACTGGGATGATATGGAGAA-3′	∼1200 bp* ∼850 bp**	
Actin-rv	5′-GCAACTGGGATGATATGGAGAA-3′		

*Genomic DNA; **cDNA.

### Cultivation of the T1 Descendants of T0 Super-Transformants

At least 100 seeds from self-fertilized transgenic plants were germinated on LS-medium containing 100 μg/mL kanamycin and 20 μg/mL D/L-phosphinothricin. Randomly selected seedlings were cultivated for 6 weeks on selective medium, and 4 weeks after the last subculture 48 plants carrying the transgenes were transferred to the greenhouse.

### Greenhouse Cultivation

Transgenic individuals, both T0 super-transformants and T1 descendants, were transferred from tissue culture 4 weeks after the last subculture, directly into 5 L pots containing peat soil (Stender AG, Schramberg, Germany). Plants were fertilized once a week using 0.5% Wuxal Super (Hermann Meyer KG, Rellingen, Germany).

### Compositional Analysis of *N. tabacum* Leaves

Dry weight was determined by oven drying (105°C, 3 h) followed by ashing in a muffle furnace (600°C, 5 h). Crude protein was determined by the Dumas combustion method using a Vario Max C/N/S-Analyzer (Elementar Analysensysteme GmbH, Hanau, Germany). Cyanophycin was measured as described in the section “CGP quantification”. AA (without Trp) were determined by HPLC (Shimadzu, Kyoto, Japan) using a cation column (LC K06, Alltech-Grom GmbH, Rottenburg-Hailfingen, Germany). AA (without Trp) were determined by HPLC (Shimadzu, Kyoto, Japan) using a cation column (LC K06, Alltech-Grom GmbH, Rottenburg-Hailfingen, Germany) according to Hackl et al. (2010). The temperature program of the column was set between 57°C and 74°C, and the pH gradient from 3.45 to 10.85. The buffer flow rate was 0.45 mL/min. AA were mixed with ninhydrin at a flow rate of 0.25 mL/min for tinting at 128°C and determined with an UV-detector at 570 nm (proline at 440 nm).”

### CGP Quantification

Cyanophycin was quantified as described by Nausch et al. (2016) which bases upon the method of Simon (1973). Three samples per individual plant of 30–35 mg of freeze-dried leaf material were homogenized with ceramic pills in a Precellys 24 homogenisator (VWR International GmbH, Erlangen, Germany) and incubated in 1 mL 50 mM Tris [pH 8.0] for 30 min. After centrifugation, the pellet was resuspended in 1 mL of 0.1 M HCL and incubated for 1 h. After another centrifugation step, 800 μL of the supernatant was used for CGP analysis. 1–10 μL of sample were filled up with 0.1 M HCL to a final volume of 800 μL and 200 μL of 5x RotiQuant Bradford reagent (Carl Roth GmbH + Co. KG, Karlsruhe, Germany) added. After 5 min incubation, samples were measured at 595 nm. A calibration curve was prepared with purified CGP from potato tubers, isolated as described by Neubauer et al. (2012). OD values of leaf samples from non-transgenic plants were subtracted from OD values of samples of non-transgenic near-isogenic control plants.

### *In vitro* CphE241 Activity Assay

In our previous study, we demonstrated that when CphE241syn is present in the plant sample, CGP is degraded during CGP quantification in the incubation step with Tris buffer and before measuring the OD at 595 nm (Ponndorf et al., 2017). Thus, we defined this protocol as protocol with active CphE enzyme. By omitting the Tris extraction step and direct incubation in 1 mL HCL, we could prevent the CGP degradation, hence in this protocol the CphE enzyme is inactivated. Comparing the results of both methods served as *in vitro* activity assay. For each individual plant, the *in vitro* activity assays was repeated 3–5 times.

### *In planta* CphE241 Activity Assay

Leaves were collected and cut along the leaf midrib into two halves in order to prepare two pooled samples from each plant. One pool was frozen immediately without homogenization to analyze the CGP content in the intact leaves at the time point of sampling (T0 sample), while the other pool was homogenized using a PT2100-Homogenizer (Kinematica AG, Littau-Lucerne, Switzerland; 30,000 rpm; 30–45 s) and incubated overnight at 22–24°C (T24 sample). Samples were freeze-dried and CGP quantified. The pools were freeze-dried and CGP quantified with at least 3–5 analysis per pool.

### Preparation of CGP-Containing HCL Extracts for Coomassie-Staining

HCL-extracts from the CGP quantification were used for Coomassie-staining. 88 μL of a 72% trichloroacetic acid (TCA) solution were added to the 800 μL HCL-extracts in order to precipitate acid soluble proteins and CGP. After incubating for 2 h at 4°C and centrifugation (16,100 *g*, 15 min, 4°C), the pellet was resuspended in 100 μL loading buffer containing 10% glycerin, 150 mM Tris [pH 6.8], 3% SDS, 1% β-mercaptoethanol and 2.5% bromophenol blue. The pH was adjusted to 6.8 with a saturated Tris solution. After denaturation at 95°C for 5 min, 1–10 μL of the samples were separated by a 12% SDS-PAGE and Coomassie-stained. PageRulerPlus Prestained Protein Ladder Mix (Thermo Fisher Scientific, Brunswick, Germany) served as marker and 5 μg CGP, purified from potato tubers, as positive control.

### Dipeptide Analysis

Dipeptides were analyzed in five samples per plant. Approximately 200 mg of dry plant material were supplemented with 1 mL of 5 mmol/L HCl, the suspensions were vortexed for 30 s and centrifuged (15,000 *g*, 10 min, 4°C). The supernatants were transferred to new 1.5 mL polypropylene tubes and lyophilized. The residues were reconstituted in 50 μL of 20% (v/v) aqueous acetonitrile and derivatized with *N*^2^-(5-fluoro-2,4-dinitrophenyl)-L-valine amide (*L*-FDVA) as described by Ehrlich and co-workers (Ehrlich et al., 2009) with modifications. In detail, aliquots of the reconstituted extracts (20 μL) were supplemented with 7 μL of water and pH was adjusted to 8.0 with 1 mol/L NaHCO_3_ using indicator paper (typically 8 μL of NaHCO_3_ were required). Afterwards, 32 μL of 36.7 mmol/L L-FDVA acetone solution were added, and derivatization of amines was accomplished during 90 min at 40°C under continuous shaking (350 rpm), before the reactions were stopped by addition of 25 μL of 1 mol/L HCl. After the change of solution color to yellow, 50 μL of acetonitrile and 114 μL of water were added, and the samples were intensively vortexed. Finally, 500 μL 0.1% (v/v) formic acid were added, the samples were centrifuged (15,000 *g*, 10 min, 24°C), and 200 μL of the supernatants were transferred to the inserts of the vials for HPLC.

Analysis of the L-FDVA DP derivates relied on the method of Antonova et al. (2019) with some modifications. In detail, 4–100 μL of each sample were loaded on a reversed phase Hypersil GOLD aQ column (100 × 1 mm, 1.9 μm particle size), installed on a Dionex Ultimate 3000 UHPLC System (Thermo Fisher Scientific, Bremen, Germany). The separations were performed at the flow rate of 150 μL/min, at 40°C in a linear gradient mode, with eluents A and B being water and acetonitrile, both containing 0.1% (v/v) formic acid. After a 2-min isocratic step at 5% eluent B, L-FDVA derivatives of the dipeptide were separated in the sequential gradients to 20, 33, and 42% eluent B in one, seven, and 4 min, respectively. After a second isocratic segment (4 min at 42% eluent B) a further gradient to 70% eluent B was run in 3 min. The column effluents were transferred on-line into a hybrid LTQ-Orbitrap Elite mass spectrometer (Thermo Fisher Scientific, Bremen, Germany), equipped with a heated electrospray ionization (HESI) source and operated in the positive ion mode. The analysis was performed under ion spray (IS) voltage of 4.0 kV, with nebulizer and auxiliary gases set to 35 and 30 arbitrary units, respectively. The capillary temperature was set to 275°C. The spectra were acquired at the mass to charge ratio (*m/z*) range of 400–2,000 and resolution of 30,000. The DP ß-*L*-aspartyl-*L*-arginine was identified in chromatograms by *m/z*, t*_R_* and co-elution with the authentic standard. Quantitative analysis relied on the standard addition approach as described elsewhere (Bilova et al., 2017). Thereby, analyte abundances were obtained by integration of corresponding extracted ion chromatograms (XICs) at specific t_R_. Peak integration was performed in Xcalibur 2.2 software.

Purified β-Asp-Arg DPs, which were used for the calibration curve, were kindly provided by the Cysal GmbH (Münster, Germany; Dr. M. Krehenbrink)^1^.

### RNA Analysis

cDNA was prepared and PCR analysis conducted as described by Nausch and Broer (2017), except that an Oligo-dT-primer was used instead of an random hexamer primer. Total RNA was isolated from 100 mg tobacco leaf tissue using Trizol reagent according to the manufacturer’s instructions (Thermo Fisher Scientific, Brunswick, Germany). RNA integrity was assessed by visualizing the 28S and 18S rRNA bands under UV light in a denaturing 0.8% MOPS-agarose gel containing ethidium bromide. For reverse transcription (RT) into cDNA, total RNA samples were incubated with DNase I at 37°C for 1 h, followed by cDNA synthesis using a commercial kit [RevertAid^TM^ H Minus First Strand cDNA Synthesis Kit (Thermo Fisher Scientific)]. 200 units of reverse transcriptase were added to 5 μg of DNase-treated RNA, 10 mM dNTP mix, 5 μM Oligo-dT-primer and incubated at 42°C for 1 h. The reaction was stopped for 10 min at 70°C. PCR was conducted with the DreamTaq DNA polymerase (Thermo Fisher Scientific). Undiluted cDNA (2 μL) were amplified in a 50 μL PCR reaction with following parameters: After an initial denaturation step (95°C for 3 min), 35 amplification cycles (95°C for 30 s, 58°C for 30 s, 72°C for 1.5 min) were carried out and completed by a final elongation step (72°C for 10 min). The PCR was performed as multiplex assay with construct-specific and actin-specific primers. The *cph*E241*syn* mRNA was detected using the primer pair CphEsyn-fw5/rv1, for *psby*-*cph*A_Te_ mRNA the primer pair PsbY-fw/CphA-rv1 was used (Table 1). Actin, as a housekeeping gene was used to compare the quantity of target mRNA/cDNA between samples and to detect contamination of samples with genomic DNA.

### Western Blot Analysis

For Western blot, crude plant extracts were prepared either from freeze-dried leaf tissue. Approximately, 200 mg plant material were added to 1 mL NPI-lysis buffer (50 mM NaH_2_PO_4_ [pH = 8.0], 300 mM NaCl, 10 mM imidazole), homogenized with ceramic pills using a Precellys 24 homogenisator (VWR International GmbH, Erlangen, Germany) and incubated for 30 min at 4°C in a shaker. After centrifugation (16,100 *g*, 4°C, 15 min) the supernatant was transferred to another reaction tube and centrifuged again. Alternatively, the Tris extract from the CGP-quantification assay was used for Western blot. The TSP concentration was quantified via Pierce^TM^ Coomassie Plus Assay Kit (Thermo Fisher Scientific, Brunswick, Germany) according to the manufacturer’s instructions. For the detection of CphE241, the Western blot was conducted as described by Nausch and Broer (2017), using an anti-His antibody as primary antibody (Nausch and Broer, 2017). TSP samples were freeze-dried and resuspended in 25 μL loading buffer containing 10% glycerin, 150 mM Tris [pH 6.8], 3% SDS, 1% β-mercaptoethanol, and 2.5% bromophenol blue. After denaturing at 95°C for 5 min, samples were separated by a 12% SDS-PAGE and electrophoretically transferred to a 0.45 μm Hybond ECL nitrocellulose membrane (GE Healthcare Europe GmbH, Freiburg im Breisgau, Germany), using a Bio-Rad *Trans-*Blot semi-dry transfer cell. 2 mA/cm^2^ were applied for 1 h using 50 mM Tris, 40 mM glycine, 0.01% SDS, and 20% methanol as the transfer buffer [pH 8.5]. Membranes were blocked with TBSTween20 (20 mM Tris [pH 7.6], 150 mM NaCl, 0.05% Tween20) and 5% non-fat milk powder for 2 h at 24°C. After three washes with TBSTween20, membranes were probed at 24°C for 2 h with a mouse monoclonal anti-His antibody (cat.: DIA 900; Dianova, Hamburg Germany) at 1:1000 dilution in Signal Boost ImmunoReaction Enhancer solution I (Merck KGaA, Darmstadt, Germany). Following another washing step, the membranes were further probed at 24°C for 1 h with a horseradish peroxidase (HRP)-conjugated donkey anti-mouse antibody (cat. no. 715-035-151; Dianova) at 1:10,000 dilution in ImmunoReaction Enhancer Solution II. Again, three washes were done with TBS without Tween20. The signals were detected by incubation in ECL chemiluminescence reagent (ECL reagent I: 1 M Tris [pH = 8.5]; 250 mM Luminol, 90 mM p-Coumaric Acid; ECL reagent II: 1 M Tris [pH 8.5], 30% H_2_O_2_) and subsequent exposure of membranes to a Kodak Biomax light X-ray film (VWR; Darmstadt, Germany) for 1–15 min. For the verification of observed differences in the CphE241 expression level, Western blots were repeated 2–3 times.

CphE241syn, produced in *E. coli* and isolated via Ni-NTA purification as described by Nausch and Broer (2017) served as positive control.

### Southern Blot

Southern blot analysis was conducted according to Hühns et al. (2008). Chromosomal DNA was digested with *Bam*HI and *Hin*dIII. The probe was amplified from the plasmid pLH7000-35s-CphE241syn using the primers cphEsyn-fw and cphEsyn-rv1 (Table 1).

### Statistical Methods

Statistical analysis was performed with SPSS, using either the non-parametric (Mann–Whitney-*U* or unpaired *T*-test) or univariate ANOVA (including the *post hoc* Bonferroni, Duncan and Tukey test). A *P*-value *P* ≤ 0.05 (two-sided) was considered to be significant.

## Results

### CphE241 Can Be Co-expressed in High Amounts in CGP-Producing Plants

**FIGURE 2:**
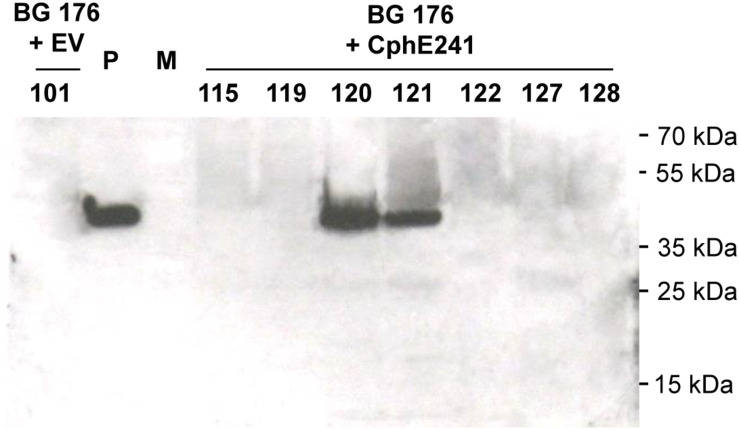
CphE241 could be detected in leaves of the *in vitro* grown events 120 and 121 by Western blot. Samples from 7 super-transformants were taken from leaves of 4 week old *in vitro* plants. 100 μg TSP per sample were analyzed in the Western blot analysis. BG 176 + EV: super-transformants, carrying the empty vector, BG 176 + CphE241: super-transformants, carrying the CphE241 encoding T-DNA. Numbers: secondary transformation events. P: 5 ng of purified CphE241syn, produced in *Escherichia coli*. M: PageRulerPlus Prestained Protein Ladder Mix (Thermo Fisher Scientific). Since the marker proteins are not conjugated with the HRP enzyme, they cannot be detected by ECL. The corresponding apparent molecular weights of the protein standards are given on the right side of the image.

Overexpression of CGP can be used to enrich plants with Asp and Arg. In two different cultivars of tobacco, 2.5% CGP/DW result in an increase of Asp and Arg of 1% DW each (Supplementary Table SM1). In order to make the AA stored in CGP bioavailable, we aimed to co-express a CGPase. For co-expression of the CGPase CphE241 in tobacco with plastidic CGP-production, we selected T2 plants of BG transformant (event) 176, described in our previous study (Nausch et al., 2016), because (1) this line carries one-copy of CGP-synthetase encoding transgene, (2) was bred to homozygosity, and (3) has a homogenous and reliable CGP-accumulation of ∼2.5% CGP/leaf DW. These T2 plants were super-transformed with the gene for CphE241syn under the control of the constitutive CaMV 35s (pLH7000-35s-CphE241syn, Figure 1), using the *bar* gene and the herbicide phosphinothricin as selection system. Putative events, transformed with the empty vector pLH7000 were named BG 176 + EV and putative events from the transformation with pLH7000-35s-CphE241syn, named BG 176 + CphE241. The presence of the transgene was verified in regenerated plants via PCR, using CphEsyn-fw5/CphEsyn-rv1 for the coding region of *cph*E241*syn*, and barPP-fw/-rv for the coding region of the selection marker (Table 1). However, via Western blot the presence of the CphE241 protein was only detected in two out of 90 super-transformants (event 120 and 121; Figure 2 and Supplementary Table SM2). In three independent clones each, the signal of CphE241 was reproducibly 2–3 times higher in event 120 compared to 121 (data not shown). All 90 events were also analyzed for the CphE241 enzyme activity, using the *in vitro* activity assay. Only in the two events 120 and 121, in which the CphE241 protein could be detected (Figure 2) was CGP degradation observed (data not shown). In order to exclude gene silencing as a reason for the absence of Cph241 in the other events, the presence of the *cph*E241*syn* mRNA was analyzed in 15 super-transformants, including event 120 and 121 as control. The mRNA could be detected in event 120 and 121 and 4 other events (data not shown).

**FIGURE 3:**
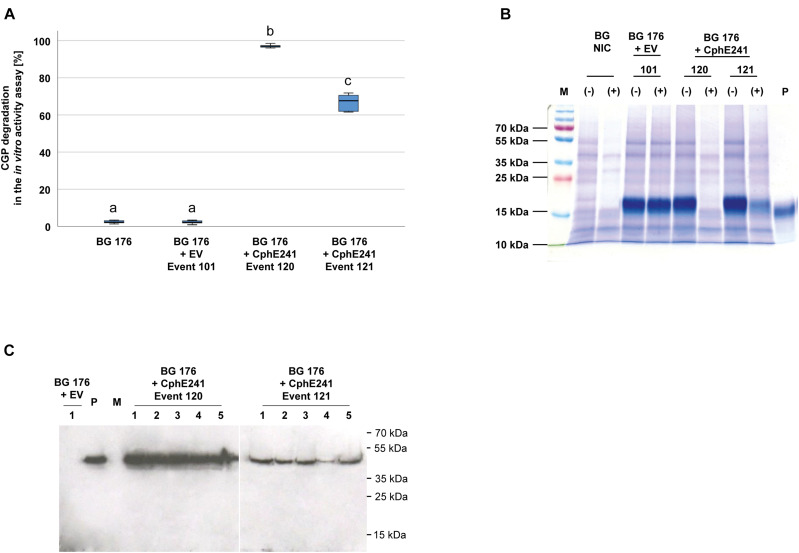
CGP degradation is almost complete in homogenized leaves from *in vitro* plants of event 120 but not in event 121. Five clones for each event were analyzed. Three fully developed, green leaves each were taken from 4 week old *in vitro* grown plants. **(A)** Box plot showing the CGP degradation in% *in vitro* in the presence of CphE241. The differences between a, b, and c are significant (ANOVA including *post hoc* Bonferroni, *p* < 0.05). Error bars represent the standard deviation; **(B)** CGP degradation visualized in a Coomassie-stained polyacrylamide gel. (–): inactivated CphE241, (+) active CphE241; P: purified CGP from transgenic potato tubers; M: marker as in **(C)**; BG NIC: near-isogenic control plants, BG 176 + EV: super-transformant, carrying the empty vector. BG 176 + CphE241: super-transformants, carrying the CphE241 encoding T-DNA; **(C)** Western blot analysis: 100 μg TSP per sample were analyzed, P: 5 ng of purified CphE241syn, produced in *Escherichia coli*. M: PageRulerPlus Prestained Protein Ladder Mix (Thermo Fisher Scientific). Since the marker proteins are not conjugated with the HRP enzyme, they cannot be detected by ECL. The corresponding apparent molecular weights of the protein standards are given on the right side of the image; 1–5 different clones of the respective events 120 or 121.

To analyze whether the individual variability between plants affects the expression of CphE241 and the capability to degrade CGP, the assay was conducted with five clones each of both events. In all clones CGP degradation occurred in a similar degree. CGP degradation was always nearly complete in all clones of event 120 in contrast to the clones of 121 (Figure 3A). CGP degradation was verified by Coomassie-staining of the samples from the *in vitro* CphE241 activity assay (Figure 3B). In Western blots, the amount of enzyme was similar in all clones of event 120 and 121, respectively (Figure 3C).

### High CphE241 Activity Was Also Observed in the Offspring of Event 120

**FIGURE 4:**
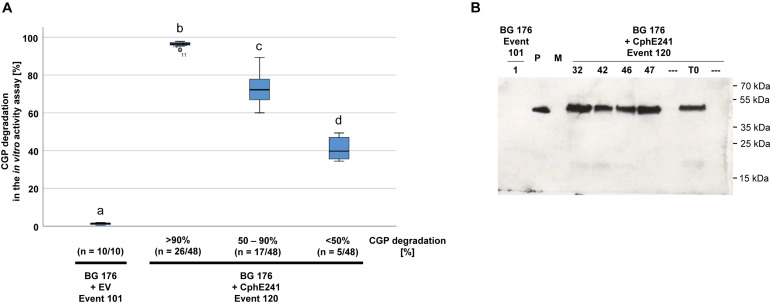
CGP degradation in the T1 offspring of event 120 can be similar to the one of the T0 parent. Forty-eight siblings were analyzed. Three samples each were taken from fully developed, green leaves of 6 week old, greenhouse grown plants. **(A)** Box plot showing the *in vitro* CphE241 enzyme activity assay. The differences between a, b, c, and d are significant (ANOVA including *post hoc* Bonferroni, *p* < 0.05). Error bars represent the standard deviation; **(B)** Western blot analysis of CphE241: 100 μg TSP per sample were analyzed, P: 5 ng of purified CphE241, produced in *Escherichia coli*. M: PageRulerPlus Prestained Protein Ladder Mix (Thermo Fisher Scientific); BG 176: primary transformant, BG 176 + EV: super-transformants, carrying the empty vector, BG 176 + CphE241: super-transformants. Since the marker proteins are not conjugated with the HRP enzyme, they cannot be detected by ECL. The corresponding apparent molecular weights of the protein standards are given on the right side of the image.

The elite event BG 176 + CphE241 event 120 was inbred in order to investigate whether the CphE241 expression will increase or decrease in the offspring. Seeds of the self-pollinated T0 super-transformants were grown on regeneration medium with phosphinothricin for the CphE241 expression cassette, kanamycin for the CphA expression cassette and cefotaxim to inhibit Agrobacteria growth. Out of the T1 offspring, 48 plants were randomly selected and the presence of the transgenes *psby*-*cph*A_Te_ and *cph*E241*syn* was verified via PCR (data not shown). All T1 siblings were transferred to the greenhouse and analyzed via the *in vitro* CphE241 activity assay in the age of 6 weeks. All plants of BG 176 + CphE241 event 120 were capable to degrade CGP (Figure 4A). In 54.2% of the individuals the enzyme degraded more than 90% of the CGP, 35.4% showed a moderate CphE241 activity with a CGP degradation ranging from 50 to 90% and only 10.4% a low CphE241 activity with less than 50% CGP degradation. The 4 T1 individuals with the highest CphE241 activity (120-32, 120-42, 120-46, and 120-47) that was similar to the T0 parent, showed a reproducibly higher CphE241 steady-state protein level in the Western blot compared to the T0 parent (Figure 4B).

**FIGURE 5:**
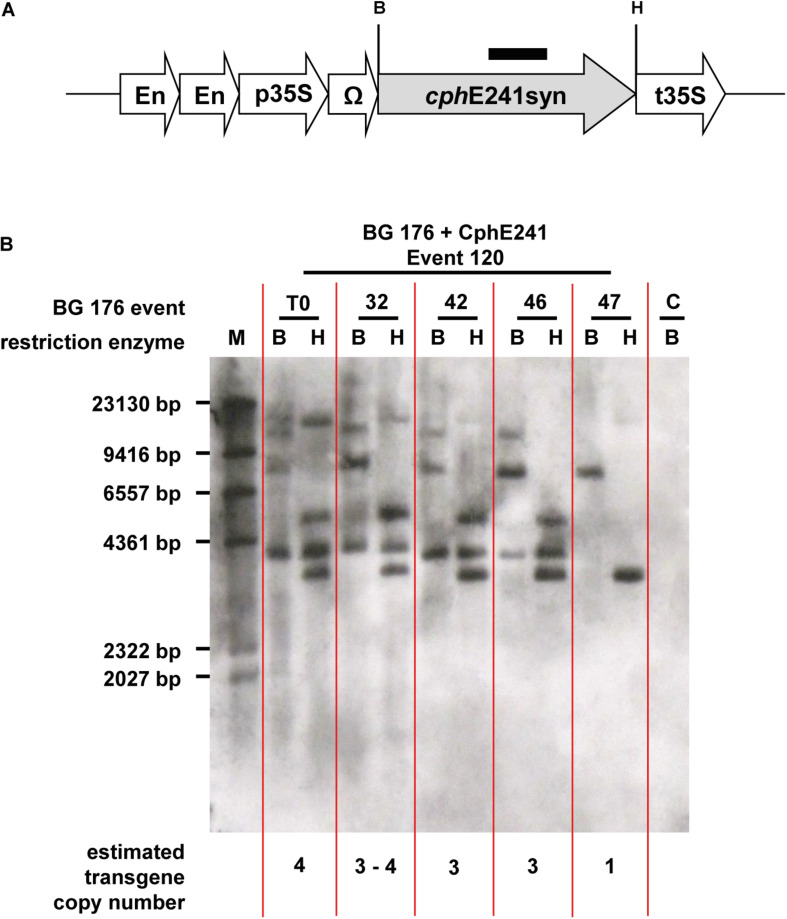
According to Southern blot analysis one transgene integration locus in event 120 seems to be decisive for high CphE241 expression. **(A)** transgene: black bar: *cphE*241syn hybridization probe; B/H: restriction enzymes sites of *Bam*HI and *Hin*dIII; *cphE*241syn: synthetic codon-optimized coding region of the cyanophycinase CphE from *Pseudomonas alcaligenes* DIP-1, including a point mutation at the amino acid position 241 of the mature protein from glycine to aspartic acid, En, Cauliflower Mosaic Virus (CaMV) 35S enhancer; p35S, CaMV 35S core promoter; t35S, CaMV 35S terminator; Ω, 5′Ω translational enhancer of the TMV. **(B)** Chromosomal DNA digested with *Bam*HI and *Hin*dIII and hybridized with the *cphE*241syn probe. M: DIG-labeled DNA Molecular Weight Marker II (Roche, Mannheim, Germany); C: BG 176 + EV event 101, T0: super-transformant of BG 176 + CphE241 event 120; 32, 42, 46, and 47: T1 descendants 120-32, 120-42, 120-46, and 120-47.

The elite event BG 176 + CphE241 event 120 and the four T1 individuals 120-32, 120-42, 120-46, and 120-47, which were selected for breeding of the T2 generation were analyzed for the copy number of the *cph*E241*syn* transgene (Figure 5A). The T0 super-transformant 120 carries at least 4 copies of *cph*E241 which are all present in the T1 individual 120-32 (Figure 5B). In the descendants 120-42 and 120-46, 3 copies were detected and in 120-47 only 1 copy was found (Figure 5B). Since all 4 T1 descendants did not differ in their CphE241 expression level, the high expression in this transgenic line might not be due to the complex integration pattern in event 120, but to the one integration locus, present in the T1 individual 120-47. This may also explain the variability in the CphE expression and CGP degradation activity observed in the T1 generation.

### CGP Is Partially Degraded in Senescing Leaves Under Greenhouse Conditions

**FIGURE 6:**
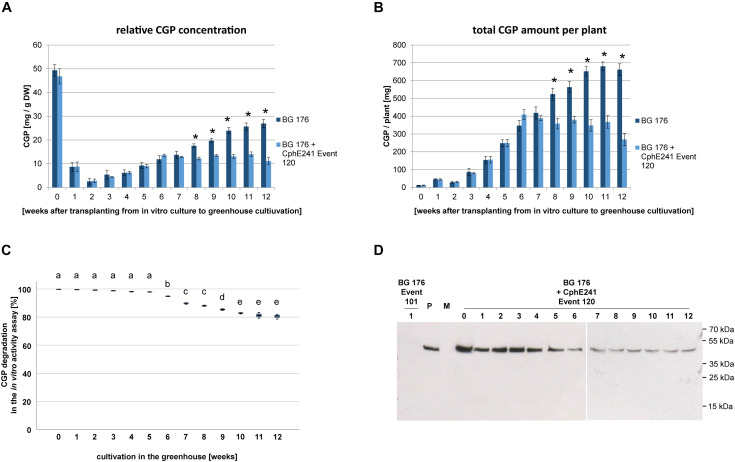
CGP accumulates in greenhouse grown clones of event 120 up to the age of 6 weeks. For each time point, the leaves of each three clones were harvested and pooled. Three to five samples per pool were analyzed. **(A)** Bar chart showing the relative CGP accumulation per DW; **(B)** Bar chart showing the absolute CGP accumulation per plant; **(C)** Box plot showing the CphE241 *in vitro* activity assay; The asterisk in **(A)** and **(B)** indicates a significant difference between BG 176 and BG 176 + CphE241 event 120. The differences between a–e in **(C)** are significant. For **(A,B)** ANOVA including *post hoc* Bonferroni (*p* < 0.05) was applied. Error bars represent the standard deviation; **(D)** Western blot analysis of CphE241: 100 μg TSP per sample were analyzed. BG 176: primary transformant. P: 5 ng of purified CphE241, produced in *Escherichia coli*. M: PageRulerPlus Prestained Protein Ladder Mix (Thermo Fisher Scientific). Since the marker proteins are not conjugated with the HRP enzyme, they cannot be detected by ECL. The corresponding apparent molecular weights of the protein standards are given on the right side of the image; BG 176 + CphE241: super-transformants, carrying the CphE241 encoding T-DNA. Numbers: 0: day of transfer of *in vitro* grown plants to the greenhouse; 1–12: weeks of cultivation in the greenhouse, *n* = 3.

Based on the CGP degradation in *in vitro* grown plants we focused on event 120 for further analysis. In order to identify the optimal harvest time (highest amount of CGP/time in the greenhouse), three independent clones each were harvested in 1 week intervals over a period of 12 weeks. In the control plant BG 176, relative CGP levels per DW and total CGP amount per plant continuously increased until week 11 (Figures 6A,B). In contrast, in the event 120, both relative (Figure 6A) and total (Figure 6B) CGP levels increased only during the first 6 weeks and remained constant from then on in the intact cells. In the same plant material, we observed a reduced CGP degradation starting from week 6 in the *in vitro* CphE241 activity assay (Figure 6C) which complies to a reduced CphE241 signal strength as shown in Western blots (Figure 6D).

**FIGURE 7:**
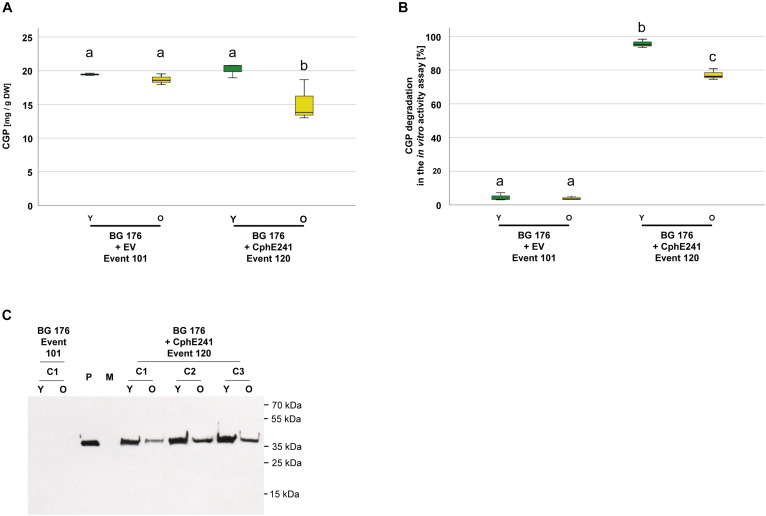
CGP degradation occurs both in green, fully developed and yellow, senescing leaves of event 120. Three clones were grown for 8 weeks in the greenhouse and fully developed, green leaves and yellow, senescing leaves sampled separately. The assay was repeated 3–5 times for each clone. **(A)** Box plot showing the CGP content, **(B)** Box plot showing the *in vitro* CphE241 activity assay; The differences in **(A)** and **(B)** between a, b, and c are significant (ANOVA including *post hoc* Bonferroni, *p* < 0.05). Error bars represent the standard deviation; **(C)** Western blot analysis of CphE241: 100 μg TSP per sample were analyzed. P: 5 ng of purified CphE241, produced in *Escherichia coli*. M: PageRulerPlus Prestained Protein Ladder Mix (Thermo Fisher Scientific). Since the marker proteins are not conjugated with the HRP enzyme, they cannot be detected by ECL. The corresponding apparent molecular weights of the protein standards are given on the right side of the image; c: clone, Y green young leaves, O: yellow old, senescing leaves, *n* = 3.

To analyze green, vital and senescing leaves separately, we grew clones of BG 176 + 7000 event 101 and BG 176 + 35-CphE241 event 120 for 8 weeks in the greenhouse and sampled vital, green and senescing, yellow leaves separately (Supplementary Figure SM1). As shown in Figure 7A, in clones of the event 101 producing only CGP, CGP levels were similar in vital and senescing leaves with 19.4 (±1.6) and 18.7 (±1.3) mg CGP/g DW, while in event 120 in old leaves CGP was significantly decreased with 15.2 (±3.1) mg CGP/g DW compared to 20.2 (±1.1) mg CGP/g DW in young leaves. The CphE241 activity assay demonstrated that the CGP degradation was reduced in older leaves (Figure 7B) in line with a reduced CphE241 signal strength (Figure 7C). β-Asp-Arg DP were significantly increased in senescing leaves of event 120 with 1445.3 (±407.8) nmol DP/g DW compared to 868.5 (±105.0) in young leaves, respectively (Table 2).

**TABLE 2:** Content of β-Asp-Arg DPs in different leaf types of the super-transformants BG 176 + 7000 event 101 and BG 176 + CphE241 event 120.

	**Young, intact leaves**	**Senescing, intact leaves**	**Young, homogenized leaves**
BG 176 + 7000 Event 101	2.95 (± 0.68)^a^	2.21 (± 0.60)^a^	1.02 (± 0.14)^a^
BG 176 + CphE241 Event 120	868.52 (± 105.04)^b^	1445.24 (± 407.82)^c^	54049.00 (± 7322.86)^d^

*Intact young and senescing and young homogenized leaves were analyzed from three independent plants (biological replicates) with five technical replicates each. β-Asp-Arg DPs were measured as [nmol DP/g DW]. The differences between a, b, c, and d are significant (ANOVA including *post hoc* Bonferroni, *p* < 0.05). Error bars represent the standard deviation of the biological replicates.*

### β-Asp-Arg Dipeptides Are Produced and Not Degraded in Homogenized Leaves

**FIGURE 8:**
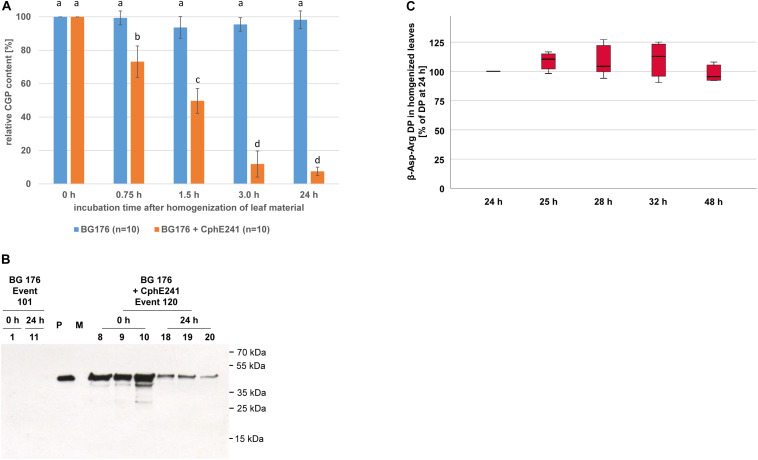
CGP degradation is almost complete 3 h after homogenization of leaves of event 120 and the β-Asp-Arg DP produced seems to be stable in the macerate. Ten clones of 6 week old, greenhouse grown plants were used for the *in planta* activity assay. The assay was 3–5 times repeated for each clone. **(A)** Bar chart showing the CGP degradation within 24 h; The differences between a, b, c, and d are significant (ANOVA including *post hoc* Bonferroni, *p* < 0.05). Error bars represent the standard deviation; **(B)** Western blot analysis of the CphE241 enzyme: 100 μg of TSP per sample were analyzed. P: 5 ng of purified CphE241, produced in *Escherichia coli*. M: PageRulerPlus Prestained Protein Ladder Mix (Thermo Fisher Scientific). Since the marker proteins are not conjugated with the HRP enzyme, they cannot be detected by ECL. The corresponding apparent molecular weights of the protein standards are given on the right side of the image; 0 h: leaf material directly after harvest, 24 h: leaf material after homogenization and incubation for 24 h at 24°C. **(C)** Box plot showing the stability of β-Asp-Arg DP in harvested, homogenized leaf material. The amount of β-Asp-Arg DPs at 24 h was set as 100%, (*n* = 6); BG 176 + EV: super-transformants, carrying the empty vector, BG 176 + CphE241: super-transformants, carrying the CphE241 encoding T-DNA, Numbers: clones. Differences between the samples were not significant (ANOVA including *post hoc* Bonferroni, *p* < 0.05). Error bars represent the standard deviation.

To assess the CphE241 activity in homogenized leaves (*in planta*), we analyzed the CGP degradation in leaf macerates. Twenty clones each of BG 176 + 7000 event 101 and BG 176 + CphE241 event 120 were grown for 6 weeks in the greenhouse and all leaves per plant were harvested. 10 of the 20 clones per plant line were directly shock frozen in liquid nitrogen (0 h). The leaves of the other 10 clones were homogenized in a commercial blender, incubated for 24 h at 24°C. Samples were taken at different time points. The amount of CGP at 0 h was set as 100%. In the BG 176 primary transformant, the CGP content was similar at 0 h and 24 h with 100% and 99% (Figure 8A), which corresponds to 18.12 (±2.17) mg/DW and 18.04 (±1.75) mg/DW, respectively. Neither obvious CGP degradation (Figure 8A) nor significant accumulation of β-Asp-Arg DPs occurred (data not shown). In event 120, CGP was only found at significant levels at 0 h with 19.34 (±2.91) mg/g DW (set as 100%), 0.75 h (73.2%), and 1.5 h (49.7%), but was almost completely degraded within 3 h with 2.16 mg (±0.74) mg/g DW (11.9%) left (Figure 8A). From then on the residual CGP level remained nearly constant and averaged at 24 h 1.32 (±0.68) mg CGP/g DW (6.8%) (Figure 8A). In parallel to the degradation of CGP, β-Asp-Arg, DPs accumulated in the homogenized tissue (24 h) with 54.05 (±7.32) μmol/g DW (Table 2), which was drastically higher than the 1.5 μmol/g DW, found in the intact senescing leaves. For complete CGP degradation 71.27 (±10.74) μmol DP/g DW are expected, while 90% degradation should result in 64.1 (±9.7) μmol DP/g DW, respectively. Thus, we measured 76.8% (±11.8) of the maximal possible amount of DP in case of complete CGP degradation and 84.4% (±9.4) of the apparently degraded CGP. The CphE241 enzyme could be detected by Western blotting in both sample types, but the level tended to be lower in the homogenized 24 h samples (Figure 8B). We could show that the DPs were stable after complete degradation of CGP at least for 48 h after homogenization (Figure 8C). The stability of DP was also demonstrated *in vitro* after addition of low amounts of purified DP (10, 50, and 250 pmol/μL which corresponded the column loads of 0.2, 1, and 5 pmol) to extraction buffer and plant extract of BG NIC plants for 1, 4, 8, and 24 h (Supplementary Figure SM2).

## Discussion

In this study, we demonstrated that the stable expression of CGPase CphE241 is sufficient to degrade 2.0% CGP/leaf DW in CGP-producing tobacco almost completely within 3 h after harvest. Although the premature degradation of CGP in intact but senescent CGPase producing plants slightly limits the CGP accumulation, this does not affect the production of β-Asp-Arg DP, since these are stable in the plants. The produced 54 μmol DP/g DW correspond to 14.7 mg DP/g DW or 1.5% DP/DW, respectively. Since Arg represents 49% of the mass of the DP, this amount is equal to 0.7% Arg/g DW. Feed for pigs and poultry, supplemented with Arg up to 1.6% DW, showed substantial effects on growth and well-being of the animals (Waseem et al., 2019). This concentration might be achieved as well when the CGPase is co-expressed in events with double of the amount of CGP. Since expression up to 9.4% CGP/DW could already be achieved (Nausch et al., 2016), this might be realistic, in case the CGPase production is sufficient.

However, under 90 events tested, there were only 2 (event 120 and 121) that produced CphE241 in a detectable amount. Based on the fact that the coding regions of both the *psby-cph*A_Te_ and the *cph*E241 coding region as well as the selection markers *npt*II and *bar* were expressed via the 35s promotor, the homologous sequences might induce a transgene silencing, which has been observed in multi-copy events (Rajeevkumar et al., 2015). Nevertheless, all 90 super-transformants produced CGP and were phosphinothricin- and kanamycin-resistant. That the *cph*E241 but neither the *psby-cph*A_Te_ gene nor the *npt*II and *bar* gene are silenced seems to be unlikely, if the 35S promoter present in all four genes causes the silencing. In addition, *cph*E241 mRNA was present in 6 out of 17 events that were analyzed via RT-PCR and event 120 with the high expression of CphE241 carries at least 4 copies of the *cph*E241 gene. It is remarkable that four events did not produce detectable levels of the protein despite the presence of the specific mRNA. Since the protein is stable for at least 24 h and the general translatability of the mRNA was proven in event 120 and 121, we can only assume that either the mRNA in the four events is somehow different from the mRNA in events 120 and 121 or the amount of *cph*E241*syn* RNA was too low to lead to detectable amounts of protein in the other events.

Analyzing the CphE241 expression under different conditions showed that the enzyme seems to be stable and active both in intact green, intact senescing and in homogenized leaves. This corresponds to our previous results where the CphE241 was as stable as the cyanobacterial CphE_al_ (Nausch and Broer, 2017). Hence there is no obvious reason for the low number of events expressing detectable amounts of CphE241. The fact, that the expression in event 121 is not sufficient to degrade the CGP in event 176 as efficient as event 120 clearly shows that a high expression is necessary for complete degradation of CGP. The high expression of CphE241 in the T0 event 120 was also transferred to its T1 offspring and seems to be associated with one integration locus, since the offspring 120-47 has only one integration locus, but a similar CphE241 expression level as in the other offspring and the T0 parent that shows a complex integration pattern. Breeding the event 120 to homozygosity of this locus might provide a pool for large-scale production of the CGP/CGPase-tobacco plants as feed.

It is remarkable that the amount of CGP did not increase in plants older than 6 weeks, if the CGPase is present in the cytosol. Under greenhouse conditions, BG tobacco shifts from vegetative growth to seed production in the age of 6 weeks (Nausch et al., 2016), and leaf senescence is expected to start (Biswal et al., 2013). Flowering and leaf senescence are associated with endoproteolytic processes in leaves and nutrient translocation from leaves to seeds (Masclaux et al., 2000; Li et al., 2016). Thus, we assume that CGP is released into the cytosol due to the decomposition of plastids in senescent tissue and degraded, if a CGPase is present. The very low amounts of DP, that were observed in intact tissue, can be explained by the occasional degradation of plastids that also occurs in green, photosynthetic active leaves, releasing plastidic CGP into the cytosol (Biswal et al., 2013). The CGP conversion in old leaves might be compensated by CGP production in young leaves, leading to constant steady state level in the whole plant.

The usage of feed that contains CGP and CGPase to provide sufficient amounts of β-Asp-Arg DPs in the digestive tract requires a very active enzyme that is able to digest CGP in a short period. We could show in this publication that the main CGP degradation occurs already within 3 h in the extract. Nevertheless, in case the plants are feed without prior extraction, the enzyme has to be stable and effective in the digestive tract, and the persistence of the feed should be sufficient to provide high amounts the CGP. Ponndorf et al. (2016) could show that CphB_Te,_ is stable enough to degrade CGP in the digestive tract of mice (Ponndorf et al., 2016). We could also demonstrate that there are no detectable differences in protein stability between CphB_Te_ and CphE241 and that CphE241 showed a drastically higher enzyme activity, both as purified proteins under *in vitro* conditions and in crude leaf extracts (Ponndorf et al., 2017). Since the stability and activity of CphE241 is equal to that of CphE_al_ (Nausch and Broer, 2017), CphE_al_ is stable in a wide range of conditions (Sallam and Steinbuchel, 2011), and CphE_al_ was found in the colon of animals (Sallam and Steinbuchel, 2009a), it might be assumed that CphE241 is also active in the intestine. Hence, it might be concluded that CGP degradation will also take place in the intestine of animals when CGP/CphE241 co-expressing fresh plants are directly fed and when the plant material is digested in the gut.

Nevertheless, tobacco is an uncommon feed. But comparing the production of CGP in tobacco and potato (Hühns et al., 2008, 2009), tobacco has the highest capacity to produce CGP without penalty on biomass (yield) and phenotype. In addition, tobacco produces high yields of leaf biomass without special demands on soil and fertilization (Billenkamp, 2010a,b, 2013), yielding up to 140–165 t leaf FW or 16–32 t leaf DW per year and hectare in a moderate climate when using mechanized farming practices (Wildman, 1979; Long, 1984; van Beilen et al., 2007). The tobacco leaves contain 20–30% protein/DW (Kung et al., 1980; Fantozzi and Sensidoni, 1983; De Jong, 1991), thus making it to an interesting feed crop for livestock. The low amounts of Arg in leaves of common tobacco cultivars (Kung and Tso, 1978; Wilkinson et al., 1981; Moldoveanu, 2005; Zeng et al., 2015) can be substituted by CGP and CGPase cooexpression as shown in this manuscript. The event 120 descendant 47 which produces sufficient amounts of CGPase carrying only one copy of the transgene can be used to transfer CGP production and degradation to nicotine-free cultivars like SL632^2^ or to the leaf-rich, gene edited “Virginia Smoking Tobacco” variety (Schachtsiek and Stehle, 2019). Since we could also show that CGP-producing BG tobacco can be ensiled and that CGP is not degraded during this process (Nausch et al., 2016), tobacco might become a valuable feed plant if β-Asp-Arg DP is also stable in the silage.

Since DPs have the potential to promote animal welfare in livestock, this approach might also be suitable for other feed crops such as cereals like barley (*Hordeum vulgare*) and maize (*Zea mays*), legumes like soybean (*Glycine max*) and pea (*Pisum sativum*), or crucifers like rapeseed (*Brassica napus*) or false flax (*Camelina sativa*). Moreover, whereas both conventional and previous biotechnological breeding efforts failed to increase (conditionally) essential AA such as Lys, Arg, Trp or Met in these plants (Ufaz and Galili, 2008), we could show in this study that the level of Arg was substantially increased via CGP/DP production. If other AA such as Lys would also be incorporated in plant-produced CGP/DP as it is the case in microorganisms (Frommeyer et al., 2014), DP would be a suitable tool to increase the underrepresented AA in these crops.

## Data Availability Statement

The datasets generated for this study are available on request to the corresponding author.

## Author Contributions

HN, AF, PW, and IB conceived the research and analyzed the data. HN, MD, and SH conducted the experiments. HN and IB wrote the manuscript. All authors contributed to manuscript revision, read and approved the submitted version.

## Conflict of Interest

The authors declare that the research was conducted in the absence of any commercial or financial relationships that could be construed as a potential conflict of interest.

## Footnotes

1 http://www.cysal.de/

2 http://www.sunchem.nl/
