# Supplementary material for: Direct Delivery of Health Promoting β-Asp-Arg Dipeptides via Stable Co-expression of Cyanophycin and the Cyanophycinase CphE241 in Tobacco Plants
Source: Front Plant Sci. 2020 Jun 19;11:842. doi: 10.3389/fpls.2020.00842 (PMC7318851; doi:10.3389/fpls.2020.00842)
Supplement: Supplementary file 1 [file Data_Sheet_1.docx]

Supplementary Material

**Figure SM1: Fully developed, green and yellow, senescing leaves of *N. tabacum* cv. Badischer Geudertheimer (BG).**

**Figure SM2: Stability of added β-Asp-Arg DP in plant extracts.** Relative amount [%] of purified β-Asp-Arg dipeptides that were added to either extraction buffer or plant extract from BG NIC plants in 3 different concentrations (10, 50, 250 pmol/µL, n = 3, ∑ n = 9) and incubated for 0, 1, 4, 8 and 24 h at room temperature. Differences between samples are not significant (ANOVA including post-hoc Bonferroni, p < 0.05). Error bars represent the standard error mean.

**Table SM1:** Crude protein and amino acid content in non-transgenic and CGP-producing Badischer Geudertheimer (BG) and Virginia Golta (VG) tobacco, described in (Nausch et al., 2016b). Since pooled samples of clones from each event were measured, there is no standard deviation. CGP: cyanophycin; NIC: non-transgenic, near-isogenic control; Event 25/24: Cyanophycin-producing tobacco events; FW: fresh weight; DW: dry weight.

|  | **BG** | | **VG** | |
| --- | --- | --- | --- | --- |
|  | **NIC** | **Event 25** | **NIC** | **Event 24** |
| Dry Weight  [% FW] | 16.28 | 18.49 | 20.22 | 21.28 |
| Crude protein  including CGP  [% DW] | 10.49 | 12.60 | 8.54 | 11.10 |
| Cyanophycin  [% DW] |  | 2.49 |  | 2.19 |
| Amino Acids  [% DW] | | | | |
| Arg | 0.40 | 1.71 | 0.36 | 1.29 |
| Asp | 1.05 | 2.07 | 0.78 | 1.59 |
| His | 0.20 | 0.19 | 0.17 | 0.17 |
| Ile | 0.31 | 0.32 | 0.28 | 0.24 |
| Leu | 0.59 | 0.60 | 0.54 | 0.48 |
| Lys | 0.46 | 0.45 | 0.40 | 0.36 |
| Met | 0.12 | 0.12 | 0.13 | 0.09 |
| Cys | 0.12 | 0.11 | 0.11 | 0.10 |
| Phe | 0.40 | 0.40 | 0.35 | 0.31 |
| Tyr | 0.21 | 0.20 | 0.19 | 0.19 |
| Thr | 0.36 | 0.35 | 0.32 | 0.29 |
| Val | 0.42 | 0.45 | 0.39 | 0.35 |
| Ala | 0.45 | 0.46 | 0.41 | 0.37 |
| Glu | 1.58 | 1.13 | 1.12 | 1.01 |
| Gly | 0.38 | 0.39 | 0.35 | 0.31 |
| Pro | 0.60 | 0.49 | 0.65 | 0.58 |
| Ser | 0.38 | 0.37 | 0.33 | 0.32 |

**Table SM2: Regeneration frequency of the transformation of *N. tabacum* BG 176-4-3, super-transformed with pLH7000 and pLH7000-35s-*cph*E241*syn*.** Calli/Explant and Shoot/Explant ratios were determined 6 weeks after transformation. n.a.: not analyzed.

| Secondary-transformant  of BG 176-4-3 | Calli/Expant  ratio  [mean/stdev] | Shoot/Expant  ratio  [mean/stdev] | No. of regenerated secondary transformants | No. of PCR positive plants | No. of plants with detectable CphE241 protein |
| --- | --- | --- | --- | --- | --- |
| pLH7000 | 1.13 ±0.28 | 0.76 ±0.12 | 26 | 26  (100%) | n.a. |
| pLH7000-35s-CphE241syn | 1.11 ±0.15 | 0.69 ±0.12 | 103 | 90  (87.4%) | 2  (2.2%) |
